# P394: Process evaluation of the sterilization of medical devices and surgical textiles in seven hospitals in Mali and Senegal

**DOI:** 10.1186/2047-2994-2-S1-P394

**Published:** 2013-06-20

**Authors:** AT Traoré, O Thioune, L Bengaly, B Ndiaye, AM Dieye

**Affiliations:** 1 CHU-Gabriel Touré , Bamako, Mali; 2 CHU-FANN et Université de Dakar , Dakar, Senegal

## Introduction

Nosocomial infections are public health concerns. In clinical procedures and chirurgical cares, sterilization is an effective procedure in the fight against these infections.

## Objectives

We evaluated the practice of sterilization process of medical devices (MD) and surgical textile (ST) in two West African countries.

## Methods

It was a prospective descriptive study conducted over a four-month period in seven hospitals in Mali and Senegal. Data were collected through interviews with supervisors of central sterilization units (CSU) and by direct observation of sterilization practices. They were entered and analyzed using Epi-info and compared to standard.

## Results

These evaluations have found that 1/7 of CSU was connected to the hospital pharmacy. Deficiencies areas were observed in 6 out of 7 CSU, leading to the conclusion that the principle of the "forward" was observed only in 1/7 of the CSU. Recommended products for pre-cleaning and disinfection were only available in 3 out of 7 CSU. Secondary packaging of MD and ST was performed only in 2/7 of the CSU. The quality assurance (QA) system doesn’t exist. Staff of 6 out of 7 CSU had never received training in best sterilization practices.

## Conclusion

The deficiencies identified led to recommendations for a better treatment of MD and ST in order to strengthen CSU and contribute to improving the quality of care and patient safety in both countries.

## Disclosure of interest

None declared

